# Role of Inflammasomes in Keloids and Hypertrophic Scars—Lessons Learned from Chronic Diabetic Wounds and Skin Fibrosis

**DOI:** 10.3390/ijms23126820

**Published:** 2022-06-19

**Authors:** Chenyu Huang, Rei Ogawa

**Affiliations:** 1 Department of Dermatology, Beijing Tsinghua Changgung Hospital, School of Clinical Medicine, Tsinghua University, Beijing 102218, China; huangchenyu2014@126.com; 2 Department of Plastic, Reconstructive and Aesthetic Surgery, Nippon Medical School, Tokyo 113-8603, Japan

**Keywords:** keloids, hypertrophic scars, NLRP3 inflammasomes, diabetic wounds, sclerosis, mechanotransduction

## Abstract

Keloids and hypertrophic scars are pathological cutaneous scars. They arise from excessive wound healing, which induces chronic dermal inflammation and results in overwhelming fibroblast production of extracellular matrix. Their etiology is unclear. Inflammasomes are multiprotein complexes that are important in proinflammatory innate-immune system responses. We asked whether inflammasomes participate in pathological scarring by examining the literature on scarring, diabetic wounds (also characterized by chronic inflammation), and systemic sclerosis (also marked by fibrosis). Pathological scars are predominantly populated by anti-inflammatory M2 macrophages and recent literature hints that this could be driven by non-canonical inflammasome signaling. Diabetic-wound healing associates with inflammasome activation in immune (macrophages) and non-immune (keratinocytes) cells. Fibrotic conditions associate with inflammasome activation and inflammasome-induced transition of epithelial cells/endothelial cells/macrophages into myofibroblasts that deposit excessive extracellular matrix. Studies suggest that mechanical stimuli activate inflammasomes via the cytoskeleton and that mechanotransduction-inflammasome crosstalk is involved in fibrosis. Further research should examine (i) the roles that various inflammasome types in macrophages, (myo)fibroblasts, and other cell types play in keloid development and (ii) how mechanical stimuli interact with inflammasomes and thereby drive scar growth. Such research is likely to significantly advance our understanding of pathological scarring and aid the development of new therapeutic strategies.

## 1. Introduction

Keloids are elevated, itchy, painful, and cosmetically disturbing fibroproliferative scars that invade the healthy surrounding skin and can significantly hamper patient psychological well-being and daily functioning. They are common in patients with an African or Asian ethnicity and, despite significant medical advances in the last decade, remain difficult to treat without recurrence. Hypertrophic scars (HSs) belong to the same fibroproliferative skin disorder as keloids and are particularly common in burn and surgery patients. They only exhibit vertical growth and generally subside after a few years; however, a joint location can lead to contractures that profoundly limit function and require surgery [1,2].

Although the heavy social and economic burden associated with these scars has spurred considerable research in the last decades, their etiological mechanisms remain to be fully elucidated, largely because of the lack of animal models. However, it is widely understood that they are driven by chronic inflammation in the reticular dermis due to failure of the wound healing process to progress past the inflammatory stage, which normally starts soon after wounding and eventually subsides [3]. The chronic inflammation augments fibroblast proliferation, myofibroblast differentiation, and their unrelenting production of extracellular matrix (ECM) [1,4].

A number of risk factors for keloids and HSs have been identified. As shown by the varying susceptibility of different ethnicities, keloids have a genetic component that strongly shapes their aggressiveness. The formation and progression of both scar types are also driven by systemic (e.g., endocrinological and metabolic) [5] and especially local factors. A key local factor is tension on the wound/scar bed due to skin stretching and underlying muscle movements: this may activate mechanoreceptors on the immune cells, endothelial cells, and fibroblasts in the ECM, thereby augmenting their pathogenic activities and scar growth. This explains why keloids have a predilection for high-tension body sites (e.g., the anterior chest) and adopt certain shapes (e.g., butterfly for anterior chest keloids), and why hypertrophic scars on joints often form contractures [1,6,7].

There is ample evidence that chronic inflammation drives pathological scar growth [3]. For example, while the keloid center is often hypoxic and exhibits relatively little inflammation, its invading edge (which experiences the strongest mechanical forces) exhibits intense inflammation. Moreover, long-term steroid plaster treatment can abolish small-moderate keloids/HSs, and adjuvant radiotherapy (which blocks angiogenesis and the consequent influx of immune factors into the wound) is indispensable for repressing keloid recurrence after surgical resection. Some of the many inflammatory factors that have been identified in keloids include cellular components such as macrophages and lymphocytes [1,8], multifunctional adhesion molecules such as CD44 [9], and chemokines/cytokines such as interleukins (ILs), transforming growth factor (TGF)-β, and tumor necrosis factor (TNF)-α [10,11,12].

Several studies suggest that normal wound healing and pathological scarring may also involve inflammasomes [13,14,15,16], which are multimeric structures that are assembled in many innate immune cell types in response to danger signals. These structures are essentially protein platforms that connect danger signal recognition to caspase-1 activation, the consequent production of the proinflammatory orchestrator cytokines IL-1β and IL-18, and their downstream proinflammatory effects [17]. These structures were first identified in 2002 and their roles in health and disease are still being elucidated [18].

Below, we will provide an overview of inflammasome structure and function, delineate the studies that show inflammasomes participate in normal wound healing, examine the few studies to date that suggest they also contribute to pathological wound healing, and then discuss the more ample research on inflammasomes in chronic diabetic wounds and systemic sclerosis (SSc), which is a skin fibrotic condition. These fields of research are relevant to pathological scarring because the chronic inflammation in diabetic wounds and the excessive production of ECM in SSc are both present in pathological scars. We will also examine studies that suggest local mechanical stimuli may crosstalk with inflammasomes. The objective of this review is to stimulate further research that could yield new therapeutic, diagnostic, treatment-monitoring, and preventive strategies for pathological scars.

## 2. Structure and Activation of Inflammasomes

Inflammasomes are created in classical innate immune cells (e.g., monocytes, macrophages, and dendritic cells) and non-classical innate immune cells (e.g., epithelial cells and myofibroblasts) by exogenous and endogenous danger signals that are, respectively, designated danger-associated molecular patterns (DAMPs) and pathogen-associated molecular patterns (PAMPs). These danger signals are recognized by pattern-recognition receptors (PRRs), five of which have been confirmed to date to assemble into inflammasomes. These PRRs include three members of the NLR (nucleotide-binding oligomerization domain, leucine-rich repeat-containing) protein family, namely, NLRP (NLR and pyrin domain-containing)-1, NLRP3, and NLRC4. The other two inflammasome-assembling PRRs are AIM2 (absent in melanoma 2) and pyrin [19,20].

**Figure 1:**
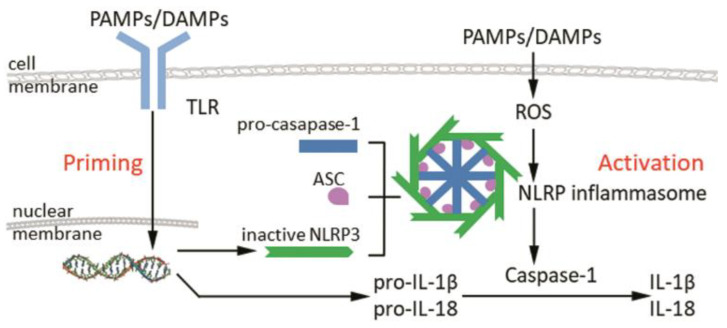
Generation of the canonical inflammasome via two sequential signaling events.

The most well-understood of these inflammasomes is the NLRP3 inflammasome. Due to this, all inflammasome studies that are mentioned in this review relate to the NLRP3 inflammasome. This structure is composed of a danger sensor (NLRP3), an adaptor protein (ASC), and an effector (caspase-1). The activity of the NLRP3 inflammasome relies on two steps, namely, the expression of its components and then their assembly into a multimeric platform. Component expression is driven by the recognition of DAMPS/PAMPs (e.g., TNF or bacterial lipopolysaccharide [21]) by PRRs such as Toll-like Receptor: these PRRs then induce the expression of NLRP3, pro-IL-1β, and pro-IL-18 via NF-κB (nuclear factor kappa-light-chain-enhancer of activated B cells) signaling [22,23,24]. The second step, inflammasome assembly, is driven by other DAMPs/PAMPs (e.g., mitochondrial dysfunction and reactive oxygen species (ROS) [23]), which are recognized by NLRP3. This causes NLRP3 to oligomerize and polymerize ASC. ASC in turn recruits pro-caspase-1 and facilitates its autoprocessing into active caspase-1. Caspase-1 then processes pro-IL-1β and pro-IL-18 into their active forms and causes the cell to lyse, thus releasing active IL-1β and IL-18 into the microenvironment [25] (Figure 1).

The activity of the NLRP3 inflammasome relies on two steps, namely, the expression of its components (priming) and then their assembly into a multimeric platform (activation). During priming, DAMPS/PAMPs (e.g., TNF) are recognized by PRRs such as TLR, which then drive component expression, with the subsequent expression of NLRP3, pro-IL-1β, and pro-IL-18 via NF-κB signaling. During activation, other DAMPs/PAMPs (e.g., mitochondrial dysfunction) drive the inflammasome assembly by active NLRP3, polymerize ASC and recruit procaspase-1, which lead to activation of caspase-1 and the subsequent cleavage of pro-IL-1β and pro-IL-18 into their active forms (IL-1β and IL-18). [Abbreviations—ASC: apoptosis-associated speck-like protein containing a caspase recruitment domain (CARD); DAMPs: danger-associated molecular patterns; IL: interleukin; NLRP: nucleotide-binding oligomerization domain, leucine-rich repeat-containing protein; PAMPs: pathogen-associated molecular patterns; PRRs: pattern recognition receptors; ROS: reactive oxygen species; TLR: Toll-like receptor.]

## 3. The Inflammasome in Normal Cutaneous Wound Healing

Normal wound healing is a tightly regulated process that involves three sequential but overlapping phases, namely, the inflammatory, proliferative, and remodeling stages. The inflammatory phase occurs within minutes of wounding and normally lasts for several days. It involves the influx of neutrophils and proinflammatory M1 macrophages, the local production of proinflammatory cytokines such as IL-1β and IL-18, and the release of growth factors that induce the recruitment, maturation, and proliferation of fibroblasts, which lay down ECM. Towards the end, it also involves a phenotypic shift in macrophages from the M1 type to the anti-inflammatory M2 type: these cells promote fibroblast activity and angiogenesis, remodel the new ECM, and suppress the inflammation [26].

There is mounting evidence that suggests NLRP3 inflammasome activity is needed for normal wound healing. First, wound healing depends on IL-1 and IL-18, which are produced by inflammasomes: both cytokines are upregulated soon after wounding [16,27], both induce collagen secretion by fibroblasts in vitro [28,29], and blockade of IL-1 receptor reduces the scarring of deep wounds in mice [30]. Second, local levels of NLRP3 protein and NLRP3 inflammasome products (cleaved caspase-1, IL-1 β, and IL-18) rise soon after punch wounding or scald burn [13,15]. Third, burn-injured mice exhibit elevated caspase-1 levels in macrophages and dendritic cells in the spleen [16]. Fourth, macrophages that are recruited into scald burn wounds were regulated by NLRP3 as a protective approach [13] and knocking out NLRP3 blocks this wound bed recruitment and prevents them from polarizing to the proinflammatory M1 phenotype that is needed for normal wound healing [13]. Fifth, mice that lack NLPR3 activity exhibit delayed wound repair, while conversely treatment of wild-type mice with a stimulatory ligand of NRLP3 accelerates punch wound healing [13,15,31]. Sixth, blocking caspase-1 activity increases the mortality of burn-injured mice [16]. Seventh, mice that lack NLRP3 activity (because NLRP3 was either blocked by treatment with an inflammasome inhibitor or knocked out) not only display decreased scald burn wound collagen production, they also have lower wound mRNA levels of proinflammatory cytokines, chemokines, and growth factors that play important roles in normal wound healing [13].

Thus, the NLRP3 inflammasome participates in normal wound healing.

## 4. The Inflammasome in Pathological Wound Healing

NLRP3 inflammasomes play important roles in physiological responses to microorganisms and other environmental stimuli that protect the host from pathogens [20,32]. They also regulate the immune homeostasis of the barrier epithelia (including the skin) [33,34]. In addition, they have also been suggested to regulate normal processes that do not relate directly to microorganisms, including the balance between erythroid and myeloid differentiation [35], normal hematopoiesis [36], hepatic metabolism [32], sterile inflammation such as that in pregnancy [37], and crosstalk with autophagy that regulates cellular integrity and prevent tumorigenesis [38]. However, similar to many other immune entities, inflammasomes can cause disease when they are dysregulated. Indeed, there is ample evidence that NLRP3 inflammasomes can drive many diseases, including pulmonary [39], hepatic [32], cardiac [40], metabolic [41], and neurodegenerative disease [42], and autoimmune conditions [43]. They can also contribute to cancer growth and metastasis [44].

**Figure 2:**
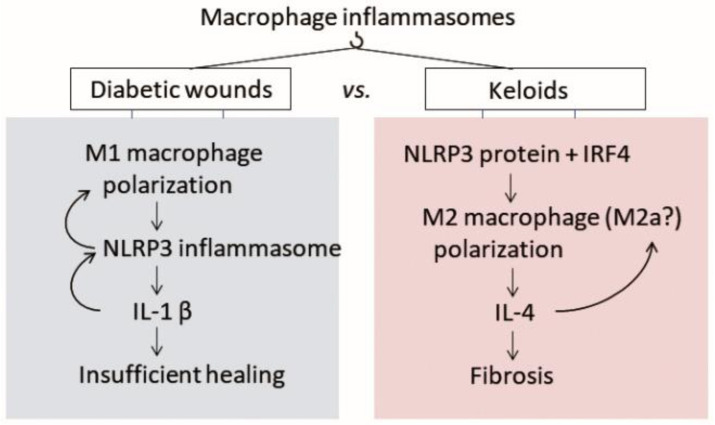
Known roles of inflammasomes in diabetic wounds and hypothetical roles of inflammasomes in pathological scarring.

Therefore, due to its importance in both health and disease, the NLRP3 inflammasome is often described as a double-edged sword [32,44,45,46]. Since there is some evidence that inflammasomes are important in normal wound healing, we propose that their dysregulation could also participate in the excessive wound healing that leads to pathological scarring (Figure 2). Below, we will discuss the few studies on NLRP3 inflammasomes in pathological scars. It should be noted that the paucity of publications on this subject most likely reflects the fact that the field is still in its infancy. Subsequently, we will discuss the more ample literature on inflammasomes in diabetic wounds and SSc.

Diabetic wounds are characterized by the inability of proinflammatory M1 macrophages to transition to M2 macrophages, which normally occurs at the end of the inflammatory wound healing phase and is needed for the downregulation of inflammation. The M1 macrophages demonstrate strong NLRP3 inflammasome activity that produces large amounts of IL-1β. This creates a feedback loop that keeps the macrophages in an inflamed state, thus leading to a wound that cannot heal. By contrast, keloids are characterized by anti-inflammatory M2 macrophages. We propose on the basis of the literature that NLRP3 protein in M2 macrophages promotes fibrosis via a non-canonical inflammasome pathway that is driven by IRF4 and produces IL-4. This process may involve a specific subtype of M2 macrophages called M2a, which are driven by IL-4, produce collagen precursors, and promote ECM formation, fibroblast proliferation, and angiogenesis. This process may also involve the inflammasome-mediated macrophage-to-mesenchymal transition (MMT) of M2 into M2 myofibroblasts that produce abundant amounts of ECM. [Abbreviations—ECM: extracellular matrix; NLRP: nucleotide-binding oligomerization domain, leucine-rich repeat-containing protein; IL: interleukin; IRF4: interferon regulatory factor 4].

### 4.1. The Inflammasome in Pathological Scarring

Lee et al. showed that compared to normal fibroblasts, keloid fibroblasts express higher mRNA levels of IL-1β and IL-18, and higher protein levels of NLRP3, its activator Notch1, the proinflammatory transcription factor NF-κB, and α-smooth muscle actin (SMA), which is a marker of myofibroblasts: these cells play a key role in the excessive production of ECM that is the hallmark of keloids. Lee et al. also found that siRNA-mediated silencing of Notch1 in keloid fibroblasts reduced their levels of the NLRP3 inflammasome components, NF-κB, and α-SMA protein. Moreover, they observed that the upregulated Notch1 expression in keloid fibroblasts was matched by reduced autophagic flux in these cells, which normally degrades Notch1: when autophagy was elevated in the keloid fibroblasts by rapamycin treatment, the Notch1 and NLRP3 inflammasome levels dropped [14].

Similarly, Vinaik et al. showed that the NLRP3 inflammasome is activated in human burn-induced keloids, as shown by their greater protein levels of cleaved caspase-1, mature IL-1β, and IL-18 relative to normal skin. The keloids were also positive for NLRP3 in their dermis on immunohistochemical staining; this was not observed in normal skin samples. Vinaik et al. also presented two lines of evidence that suggest that the upregulation of the NLRP3 inflammasome in burn-induced keloids may reflect altered local immunometabolic responses. First, burnt skin and especially keloids exhibited aberrant glucose metabolism. Second, treating human burnt skin with shikonin, a glycolysis inhibitor, reduced NLRP3-mediated inflammation and cleaved caspase-1 and mature IL-1β levels in vitro [47].

Finally, Do et al. showed that keloid tissues express higher caspase-1 and IL-18 levels than normal skin [48].

Thus, pathological scars demonstrate upregulation of NLRP3 inflammasomes.

### 4.2. The Inflammasome in Chronic Diabetic Wounds

Similar to pathological scars, chronic diabetic wounds are characterized by chronic inflammation, although the outcome is insufficient healing and failure to close rather than the excessive ECM deposition seen in pathological scars. Human diabetic wounds express higher levels of NLRP3, caspase-1, and IL-1β mRNA and protein than nondiabetic wounds [49], which suggests that inflammasomes may participate in the poor healing of diabetic wounds.

Several lines of evidence then suggest that in particular, inflammasomes in proinflammatory M1 macrophages may play a vital role in diabetic wounds, as follows. First, macrophages isolated from human and murine diabetic wounds exhibit high inflammasome activity [50]. Second, there is a growing body of research that suggests that (i) diabetic wounds do not undergo the normal shift from M1 to anti-inflammatory M2 macrophage polarization during the inflammatory phase of wound healing and therefore contain high and sustained M1 macrophage numbers [26,51,52,53], and (ii) this inability to shift from the M1 phenotype reflects the high circulating glucose levels in diabetes, which is an exogenous danger signal. Indeed, when normal macrophage cells are treated in vitro with high glucose (138.8 mmol/L), they acquire the M1 phenotype [54]. Zhang et al. showed that such cells also exhibit elevated NLRP3 and IL-1β mRNA and protein levels [49]. Moreover, Dai et al. found that rapamycin treatment (which induces the autophagic responses that downregulate NLRP3 inflammasomes) inhibits high glucose-induced NLRP3 inflammasome activation in a macrophage cell line [55]. Third, agents that directly or indirectly inhibit inflammasome activity improve diabetic wounds [56], and this associates with M1 to M2 macrophage switching. Thus, when punch wounds in rats are treated with the indirect NLRP3 inflammasomes inhibitor of metformin (which lowers glucose levels), healing is accelerated in association with lower NLRP3 inflammasome activation and greater polarization to M2 macrophages [57]. Similarly, treatment with agents that directly (e.g., glyburide or BAY 11-7082) inhibit NLRP3 inflammasome in macrophages improves wound closure in mice with genetic [58] or streptozotocin-induced diabetes [59] in conjunction with reduced active caspase-1 and IL-1β levels and switching of M1 macrophages to M2 macrophages in the wound site [50,56].

Thus, diabetic wounds exhibit NLRP3 inflammasome activity in their macrophages, and this activity contributes to the poor healing of these wounds.

Since the IL-1β in the medium from cultured diabetic wound biopsies activates NLRP3 inflammasomes in bone marrow-derived macrophages, the elevated inflammasome activity in diabetic wounds may induce a positive proinflammatory feedback loop that operates on macrophages [50] (Figure 2). There is also evidence that the inflammasome in diabetic wound macrophages delays diabetic wound healing by modulating neutrophils in a proinflammatory feed-forward loop. Thus, neutrophils are normally recruited early to wounded skin to clear microorganisms, which they achieve by releasing their nuclear and granular contents into the microenvironment via web-like chromatin structures called neutrophil extracellular traps (NETs) [60]. The neutrophils then die via NETosis, which is a unique form of cell death. In diabetic wounds, however, neutrophils are more prone to NETosis; this may contribute to the poor wound healing [61,62]. This high NETosis activity may reflect the high levels of IL-1β and IL-18 that are released into the wound bed by the macrophage inflammasomes: IL-1β and IL-18 are known to upregulate NET formation and prime neutrophils for NETosis, respectively [63]. The NETs then further activate macrophage inflammasomes, leading to a self-perpetuating cycle of inflammasome and NET production that may contribute to the chronic inflammation observed in diabetic wounds. Indeed, when the NLRP-3 inflammasome-NET axis was inhibited with an exogenous growth factor (MFG-E8), the healing of diabetic wounds was accelerated [63].

#### 4.2.1. Macrophage Polarization Is Also Disturbed in Keloids, but in the Opposite Direction

Diabetic wounds are characterized by high frequencies of M1 macrophages and their failure to progress to the anti-inflammatory M2 phenotype. By contrast, recent studies suggest that pathological scarring associates with high frequencies of M2 macrophages [64,65]. Thus, these studies report that (i) biopsies of human surgical wounds taken immediately and 3 h after wounding show that later HS formation associates with low early M1 cytokine (e.g., IL-6) levels [66], (ii) keloids have higher frequencies of M2 macrophages at their leading edge than normal skin and scars [8], and (iii) the proliferative phase of HSs associates with excessive infiltration of M2 cells [67].

Since inflammasome activation plays a well-known and key role in M1 macrophage polarization [68], the M2 predominance in pathological scars suggests that the inflammasome cannot participate in pathological scarring. However, the evidence to date (described in Section 4.1) suggests that the NLPR3 inflammasome is definitely activated in keloids. What role could the inflammasome be playing in pathological scarring?

There are a number of possibilities. First, Liu et al. showed that asthma also associates with both NLRP3 inflammasome upregulation and M2 polarization. Thus, they reported that: (i) compared to control subjects, patients with asthma demonstrated a lower M1/M2 ratio and higher NLRP3, ACS, caspase-1, and IL-1β levels in their peripheral blood monocytes; (ii) treatment of control and patient monocytes with siRNA against NLRP3 downregulated IL-4 expression (but not TNF-α or interferon-γ expression) and reduced M2 monocyte frequencies (i.e., the M1/M2 ratio rose), whereas siRNA against ASC or caspase-1 had no such effect; and (iii) when NLPR3 was deleted, mice with allergic asthma showed downregulated IL-4 secretion and higher M1/M2 ratios in the lungs and less lung inflammation. Further experiments then showed that similar to in human monocytes, asthma in mice is driven by NLRP3 but this relationship does not involve ASC or caspase-1. Rather, it is mediated by binding of the M2-promoting transcription factor IRF4 (interferon regulatory factor 4) to NLRP3, which then induces M2 polarization [69]. This unusual role of NLRP3 is supported by a recent study that showed that when NLRP3 is stabilized and therefore protected from proteasomal degradation, it associates with IRF4 and results in M2 polarization both in vitro and in vivo [70]. It is possible that NLRP3 plays this role in keloids as well since IL-4 has been suggested to initiate and perpetuate skin fibrosis [11,71]. Thus, rather than contributing to the canonical M1-associated functions that participate in diabetic wounds, NLPR3 could play hitherto unknown roles in pathological scars. This is supported by the fact that NLPR3 can also form non-canonical inflammasomes by associating with caspases other than caspase-1 (caspase 11 in mice and caspase 4/5 in humans [72,73]).

Second, M2 macrophages are not homogeneous: they can be categorized into four subgroups according to the signals that generate them and their biological functions [65]. Thus, M2a are activated by IL-4 or IL-13, produce collagen precursors, and promote ECM formation, fibroblast proliferation, and angiogenesis. M2b are elicited by IL-1 receptor ligands or toll-like receptors (TLRs) and have a regulatory role: they suppress inflammation via IL-10 and produce metalloproteinases. M2c are activated by IL-10, TGF-β, and glucocorticoids and remodel the ECM. M2d are generated by IL-6 and adenosine and inhibit M1 macrophages. This M2 diversity suggests that pathological scarring could possibly be due to excessive frequencies of M2a macrophages, or a combination of subsets, or even a hitherto unknown macrophage phenotype. Since the role of inflammasomes in these subtypes is unknown, it would be of interest to determine whether inflammasomes in a particular M2 subset participate in pathological scarring (Figure 2).

Finally, the studies in Section 4.1 show that fibroblasts from pathological scars clearly exhibit inflammasome upregulation (note that the inflammasome levels in macrophages from pathological scars have not yet been reported). As will be detailed below in the section on SSc, the inflammasomes in fibroblasts strongly promote fibrosis, which is the hallmark of pathological scarring. Thus, it is possible that the inflammasome plays pathogenic roles in both diabetic wounds and pathological scars by derailing the normal activities of different cell types, namely, macrophages in diabetic wounds (which cannot shift to the M2 phenotype) and fibroblasts in pathological scars (which undergo excessive myofibroblastic differentiation and become highly profibrotic). This may explain why the chronic inflammation in diabetic wounds and pathological scars has different outcomes, namely, insufficient wound healing in the former and excessive ECM deposition in the latter.

### 4.3. The Inflammasome in Skin Fibrosis

Similar to other fibrotic conditions in the skin and other organs, pathological cutaneous scars exhibit excessive accumulation of fibroblasts and ECM (mainly collagen type I). Several lines of evidence suggest that the inflammasomes in local fibroblasts participate in skin fibrosis by inducing these normally quiescent cells to differentiate into pathogenic myofibroblasts, which produce high levels of ECM. Artlett proposes that such inflammasome activation in these non-immune cells initiates skin fibrosis and is followed by inflammasome activation in immune cells that amplify local inflammatory responses [74].

SSc is characterized by pronounced collagen secretion by activated myofibroblasts that results in progressive fibrosis of the skin and visceral organs [75,76]. Artlett’s group showed that cutaneous SSc dermal fibroblasts overexpress 40 genes that associate with inflammasomes or their downstream signaling molecules, including NOD2 (nucleotide-binding oligomerization domain containing 2), NLRP3, AIM2, IL1B, and IL18. Moreover, these fibroblasts exhibit increased secretion of IL-1β and IL-18. This group also showed that mice that lack NLRP3 are resistant to a model of SSc: thus, while subcutaneous injections with bleomycin induce excessive collagen deposition that results in marked skin thickening and severely compromised lung architecture in wild-type mice, NLRP3-/- mice display almost no skin thickening and the lung architecture is normal. Similarly, when fibroblast lines from wild-type and NLRP3-/- mice are treated with bleomycin, the knockout lines secrete less hydroxyproline. Thus, continuous inflammasome activation may mediate the myofibroblast differentiation and fibrosis that characterizes SSc [77].

In summary, a skin fibrotic condition associates with inflammasome activation in (myo)fibroblasts that mediates the disease profile.

#### 4.3.1. The Inflammasome in the Epithelial-to-Mesenchymal Transition (EMT)

Myofibroblasts can be the result of EMT, which is a hallmark of fibrotic diseases [78]. There are several lines of evidence showing that keloids also exhibit signs of EMT. First, keloids express high levels of TNF-α and TGF-β and normal keratinocytes cultured with these cytokines causes them to gain a fibroblastic morphology, express the mesenchymal marker vimentin [79]. Second, keloids are characterized by hypoxia and stimulating keloid-derived keratinocytes with hypoxia-inducible factor (HIF)-1α in vitro induces them to develop a fibroblast-like appearance and enhanced invasiveness [80]. Third, the epidermis of keloid tissues has significantly more vimentin+ cells than the epidermis of normal skin. Moreover, these vimentin+ cells are located in the reddish inflamed invading edge of the keloids [79]. Similarly, epithelial cells from keloid tissues express vimentin and another mesenchymal marker (fibroblast-specific protein 1) and lack E-cadherin expression [81]. Thus, keloids exhibit EMT.

Interestingly, there is also a recent study showing that when keratinocytes are exposed to low hydration (by increasing the extracellular concentration of sodium), their expression of caveolin-1 drops, which in turn increases the transcription of Snail, a transcription factor that represses E-cadherin transcription. This is significant because reducing hydration promotes EMT in keratinocytes. Thus, this study links poor wound hydration, which is well-known to promote hypertrophic scarring, to EMT via a caveolin-1-Snail-E-cadherin axis [82].

Many studies on liver, kidney, lung, and intestine fibrosis show that the NLRP3 inflammasome can promote EMT during organ fibrosis by either or both of two mechanisms. In the first, inflammasome-derived IL-1β upregulates TGF-β, which is a known trigger of EMT. In the second, inflammasome oligomerization and activation is not needed: rather, EMT is triggered by the receptor in NLRP3, which enhances the activity of the R-Smads that are activated by TGF-β [24,83]. Thus, it is possible that the inflammasome also drives the EMT that leads to excessive ECM production.

#### 4.3.2. The Inflammasome in the Endothelial-to-Mesenchymal Transition (EndoMT)

Inflammasomes can also participate in EndoMT, thereby promoting fibrosis. This is shown by in vitro and in vivo models of mechanical ventilation-induced pulmonary fibrosis, which both exhibit NLRP3 activation. Thus, when primary murine lung endothelial cells are stretched, they convert via EndoMT to myofibroblasts, as shown by their loss of vascular endothelial-cadherin and CD31 expression and gain of α-SMA and vimentin expression. Knockout of NLRP3 abrogates this effect. Similarly, mechanically ventilated mice display less pulmonary fibrosis when NLRP3 is knocked out [84].

Studies on patients with SSc and mouse models of this disease show that cells undergoing EndoMT can be detected in the dermal vessels [85]. The vascular endothelium in keloid tissues also contains cells that co-express both vimentin and CD31 [86]. However, whether the inflammasome participates in EndMT in SSc and keloids remains unclear.

#### 4.3.3. Macrophage-to-Myofibroblast Transition (MMT)

Macrophages are highly plastic cells: for example, they can differentiate into endothelial-like cells and adipocytes [87,88]. A recent study showed that macrophages can also differentiate into myofibroblasts via a process called MMT and that in fact two-thirds of fibroblasts in cutaneous punch wounds are derived from cells of myeloid origin [89]. MMT has been shown to contribute to subretinal fibrosis secondary to age-related macular degeneration (20–30% of the infiltrating macrophages co-express α-SMA) [90] as well as renal fibrosis [91]. Interestingly, the MMT-derived myofibroblasts in renal fibrosis bear an M2 phenotype, which suggests that they are specifically derived from this macrophage subtype. Selective depletion studies with the obstructed kidney model show that these cells are major contributors to renal fibrosis [91]. Thus, M2 macrophages can play a pathogenic profibrotic role by converting into myofibroblasts. Given the predominance of M2 macrophages in pathological scars and the prominent role of myofibroblasts in these scars, it would be of interest to determine whether MMT also participates in pathological scarring.

Since the role of inflammasomes in MMT has not yet been studied, it would also be of great interest to explore whether NLRP3 or other inflammasomes participate in MMT, especially in wounds.

### 4.4. Crosstalk between the Inflammasome and Local Mechanical Stimuli

Mechanical stimuli have been implicated in soft tissue fibroses [92,93,94], including mechanical ventilation-induced pulmonary fibrosis [84]. The mechanism underlying these effects is called mechanotransduction, which is where cells sense extracellular mechanical forces, transduce these signals, and thereby change their intracellular biochemistry and gene expression. A key sensor of these mechanical stimuli is the cytoskeleton [95,96].

As detailed in a recent review, the NLRP3 inflammasome activation can be regulated by the mechanical environment, thereby contributing to tissue fibrosis [97]. The evidence for this includes several studies that show that NLRP3 inflammasome activation associates with actin polymerization [98], the presence of filamentous actin [99], actin rearrangement [100], and the presence of vimentin, an intermediate filament that plays a critical role in stabilizing intracellular architecture [101]. The lung ventilation-induced fibrosis experiments with NLRP3-knockout mice that were described above also directly link mechanical stretch to inflammasome activity [84]. Moreover, EMT and EndoMT, which both involve inflammasomes, are also upregulated by mechanical signals [102,103].

**Figure 3:**
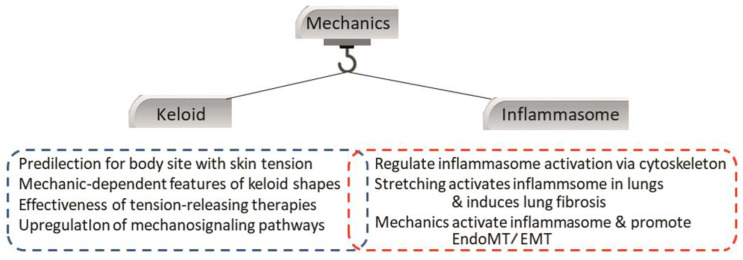
Mechanical stimuli may activate the NLRP3 inflammasome, thereby contributing to the fibrosis that leads to pathological scarring.

As mentioned above, local mechanic stimuli are a key risk factor of keloids. This is supported not only by the predilection of keloids for high-tension body sites and body region-specific growth patterns [104] but also the fact that tension-releasing surgical techniques (e.g., incision design and suturing method) greatly reduce recurrence rates after keloid excision [105,106]. Moreover, mechanical stimulation of keloids associates with enhanced growth. For example, when the symmetrical butterfly-shaped anterior chest keloid of a patient was accidentally bisected at the midline, the butterfly became asymmetrical over time: due to the right-handedness of the patient, the right side kept growing strongly, whereas the growth of the left side stalled and signs of improvement were observed [107]. In addition, mechanosignalling pathways (e.g., the TGF-β/Smad pathway) are upregulated in cutaneous scar cells, particularly myofibroblasts [108,109]. Finally, it was observed that treatment with the ECM protein asporin, which is highly expressed in keloids, alters mechanosignaling in human dermal fibroblasts, thereby blocking their maturation and ability to remodel the ECM [92] (Figure 3).

Both keloids and the NLRP3 inflammasome are mechanosensitive. The mechanosensitivity of keloids is displayed by their predilection for high-tension body sites, their growth in the direction of prevailing skin tension, the effectiveness of tension-releasing therapies (e.g., Z-plasties and local flaps), and the upregulation of intracellular mechanosignaling pathways. The mechanosensitivity of the NLRP3 inflammasome is indicated by the ability of cytoskeletal changes to activate this immune entity. It is also upregulated in fibrotic conditions that are induced by stretching, and this associates with the profibrotic EndoMT. [Abbreviations—EndoMT: endothelial-mesenchymal transition; NLRP: nucleotide-binding oligomerization domain, leucine-rich repeat-containing protein].

Notably, Butzellar et al. showed recently that the high-tension body sites that are prone to keloid formation have lower frequencies of M1 macrophages and similar numbers of M2 macrophages compared to low-tension sites [110]. This further supports the notion advanced above that M2 macrophages may play an important role in pathological scarring. This together with the findings reported in Section 4.2.1 and Section 4.3.3 suggest that that NLRP3 in M2 macrophages could promote pathological scarring by two mechanisms: (i) NLRP3-mediated M2 cytokine production (particularly IL-4), which is known to convert human dermal fibroblasts into proliferative and ECM-producing fibroblasts [111]; and/or (ii) conversion of M2 macrophages into M2 MMT-derived myofibroblasts that are highly fibrotic. The latter possibility is supported by the fact that inflammasomes participate in both EMT and EndoMT.

## 5. Future Directions

**Figure 4:**
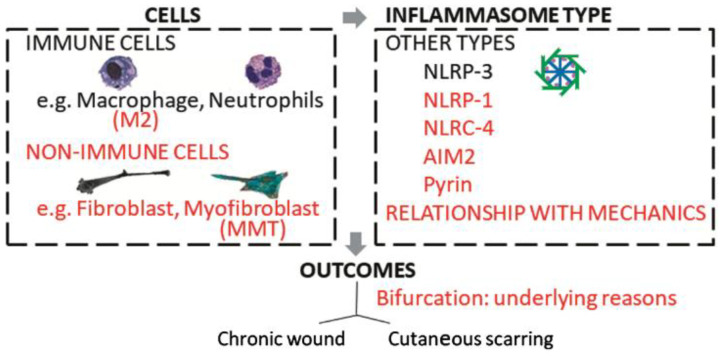
Future directions in research on the roles of inflammasomes in pathological scarring.

The studies described above show that although there is still a paucity of studies, inflammasomes appear to be upregulated in pathological scars. Our subsequent literature review then suggested that inflammasomes could potentially drive pathological scarring by causing M2 macrophages to adopt pathogenic activities, namely, producing M2 cytokines via a non-canonical NLRP3 pathway and/or undergoing MMT, both of which could lead to the heavily fibrotic myofibroblast behavior that is the hallmark of pathological scars. We also speculate that mechanical tension could drive either or both of these putative inflammasome-mediated activities. While these possibilities remain highly speculative, the intention of this review is to prompt more research on how inflammasomes, including non-canonical NLRP3 and other inflammasomes, participate in normal and abnormal wound healing. It will be of particular interest to determine how inflammasomes act differently in keloids and diabetic wounds, thus driving them towards the totally opposite outcomes of excessive and insufficient wound healing, respectively (Figure 4). This research could help lead to the development of effective therapeutic, diagnostic, and preventive strategies against pathological scarring.

What is currently known is marked in black: thus, NLRP3 and immune cells such as macrophages and neutrophils may participate in pathological scarring. What should be researched to improve the field is marked in red: what is the role of M2 cells in keloids? Is the inflammasome also important in non-immune cells such as (myo)fibroblasts? Does MMT link M2 macrophages, inflammasomes, and myofibroblastic activity? Can non-canonical inflammasomes contribute? How do these elements relate to mechanical signaling? [Abbreviations—AIM2: absent in melanoma 2; MMT: macrophage-to-myofibroblast transition; NLRP: nucleotide-binding oligomerization domain, leucine-rich repeat-containing protein]. Research on the role of inflammasome-induced myofibroblast formation in pathological scarring is likely to be particularly rewarding because myofibroblasts act as inflammatory cells as well as structure-creating cells [112]. Several studies have shown that targeting the inflammasomes in myofibroblasts is a viable anti-fibrotic strategy. Thus, Cáceres et al. showed that treating cardiac myofibroblasts with a cocktail of TGF-β1, lipopolysaccharide, and ATP (T+L+A) promotes NLRP3 inflammasome priming and activation, myofibroblast differentiation, and increased collagen deposition while treatment with serelaxin abrogates all of these effects. They then observed that serelaxin treatment in vivo reduced isoproterenol-induced cardiomyopathy along with interstitial TGF-β1 levels and NLPR-3 inflammasome activity in the ventricle [113]. Pinar et al. also showed with a dermal fibroblast line that relaxin treatment abrogated the ability of T+L+A to induce NLRP-3 inflammasome priming and activation, myofibroblast differentiation, and collagen, IL-1β, and pro-IL-18 production. Interestingly, silencing of caspase-1 nullified these effects of relaxin, which suggests relaxin can directly modulate the myofibroblast NLRP3 inflammasome at the level of caspase-1 [25]. Studies on the functions of inflammasomes in myofibroblasts in pathological scars are thus warranted.

## 6. Conclusions

There is some (still sparse) evidence that inflammasomes may drive the abnormal inflammation and excessive accumulation of ECM that characterizes pathological cutaneous scarring. These effects may be mediated by crosstalk between inflammasomes and mechanotransduction pathways, perhaps via the cytoskeleton. Further systematic research on different inflammasome types in macrophages, myofibroblasts, and other cells, and the role of mechanical stimuli, is warranted. Such research could reveal new avenues in terms of keloid etiology and therapeutic strategies.
